# Shifting away from the business-as-usual approach to research conferences

**DOI:** 10.1242/bio.056705

**Published:** 2020-10-23

**Authors:** Chelsie W. W. Counsell, Franziska Elmer, Judith C. Lang

**Affiliations:** 1 Department of Biology, Fairfield University, 1073 North Benson Road, Fairfield, CT 06824, USA; 2 Independent Researcher, Sagenbachstrasse 17, 6442 Gersau, Switzerland; 3 Atlantic and Gulf Rapid Reef Assessment, PO Box 35, Ophelia, VA 22530, USA

**Keywords:** Virtual conference, Coral reefs, Climate change

## Abstract

To combat the climate crisis, we need rapid, unprecedented social change. Scientists can play a lead role by signaling to society that we recognize the critical importance of redesigning our business-as-usual approach to research conferences. Traditional research conferences have high CO_2_ emissions as well as significant financial and travel time costs for participants. Using available technology, early career scientists Chelsie Counsell and Franziska Elmer created a global, virtual, coral reef research conference with live talks, recorded contributions, and networking events. Funding from The Company of Biologists allowed this event to be free, supporting attendance of 2700 subscribers and content contributions from 165 participants from diverse backgrounds and career stages. We provide metrics on content viewership and participation in networking activities, note the success of incorporating regionally focused sub-events, and discuss the emergence of a collaborative research project. We highlight the broad accessibility of virtual conferences as well as their increased flexibility in programming, health benefits, and cost savings. Our approach to organizing and hosting a global, low-carbon emission research conference is documented. Finally, we propose a hybrid approach to future conferences with virtually connected remote (sub-regional or local) hubs.

## Abstract (precis)

**Summary:** This meeting review provides details on a potential future direction for scientific conferences that incorporates restrictions relevant to the current pandemic and climate change.

## Introduction

Research conferences provide opportunities to share science, network, and reach innovative solutions through synthesized ideas. We discover collaborators, obtain positions, present our research, and feel inspired by plenary talks. Conference attendance is considered vital for the growth of scientific fields and for the career trajectory of most scientists. Unfortunately, the traditional approach to research conferences, often involving travel over great distances (including intercontinental flights), can have a large CO_2_ footprint. Global Coral Reef Week (GCRW) was designed to rethink this paradigm by organizing a global coral reef research conference with low CO_2_ emissions and high accessibility.

The average amount of CO_2_ emitted from travel to a research conference has been calculated at ∼800 kg per presenter (Spinellis and Louridas, 2013). Long-distance travel is particularly problematic: a seat on a one-way intercontinental flight is estimated to release more CO_2_ (3000 kg) than the average annual emissions for one person living in Britain or ten people living in Ghana (Klöwer et al., 2020). We know the climate crisis is real. Its effects occur globally through increased risk of deadly heat waves (Mora et al., 2017), intensified weather patterns, land degradation, increased food insecurity (IPCC, 2019a), loss of ice sheets and glaciers, marine heat waves, and ocean acidification (IPCC, 2019b). Coral reef scientists personally experience the high costs of climate change, as our study sites bleach with an increasing frequency in overly warm oceans, such that their continued existence is threatened (IPCC, 2019b).

To reduce the effects of climate change, we need to stop burning fossil fuels (IPCC, 2019a). The climate crisis is not a technological or scientific problem. The climate crisis is a problem of inadequate willpower, resulting from capitalism and institutionalized definitions of growth and success at all levels, from the global community to the individual (Jewell and Cherp, 2019). Scientists cannot save coral reefs through research alone. We *can* lead by example, through rapid shifts in our collective actions by redesigning our business-as-usual approach to both our professional and personal lives. We should be part of the energy that drives necessary, unprecedented social change.

International, in-person coral reef conferences are intended to help study and save coral reefs, but, paradoxically, the long-distance air travel of participants to these events directly contributes to their demise (Nevins, 2014). GCRW was designed to pilot an approach to research conferences that removes long-distance flights while retaining the ability to network and share science. This dramatically reduces conference CO_2_ emissions and increases conference accessibility (Hiltner, 2019). We originally envisioned a collection of inexpensive, virtually-connected, remote meeting ‘hubs’ organized at local and sub-regional scales. This approach would blend in-person interactions with access to live-streamed plenary talks (including interactive questions and answers) and include the curation of a digital content repository. Our vision was enthusiastically received by colleagues, particularly in the Caribbean, Brazil, Hawai‘i, the continental US, and parts of Europe.

From January to March, we confirmed local hosts for remote meeting hubs in 16 locations. When COVID-19 then restricted face-to-face meetings and group gatherings, we pivoted to create an entirely virtual conference. GCRW thus became an opportunity for coral reef scientists to share their research, network with new colleagues, and try the virtual conference experience.

## Conference components and metrics

When organizing GCRW, we strived to retain the key components of an in-person conference, i.e., research talks, plenary talks, workshops, networking, and other social events. Contributed talks were uploaded as recorded presentations to a GCRW conference YouTube channel divided into nine topical playlists. Our conference website included advice on how to use freely available software to create a digital recording of a presentation and tips for producing engaging science communication videos. The 108 research talks that were contributed from participants across the globe featured reefs in the Atlantic, Indian, and Pacific Oceans, as well as the Caribbean, Mediterranean, and Red Seas. Early career scientists and students submitted over 45% of the presentations. Reef scientists, resource managers, and coral reef enthusiasts submitted the remainder. On the final day of the conference, recorded research presentations had an average of 101 views per video. One month after the end of GCRW, this average had increased to 165 views per video for a total of over 17,500 views.

For our ten live-streamed plenary talks, we invited a diverse group of speakers who collectively represented seven countries, five of whom were within eight years of their PhD (two within two years). Plenary talks were divided into five thematic sessions that attendees could join via Zoom to participate in questions and answers. All plenary talks were also live-streamed on our YouTube channel, and the recordings are digitally archived on our website. An average of 141 attendees watched the plenaries live, with an average additional 281 views on YouTube at the end of GCRW.

After COVID-19 restrictions forced the shift to an entirely virtual format, the local hosts for three hubs requested virtual support to live-stream regionally focused research talks and discussions. We therefore added live-streamed sessions for Brazil (four speakers, hosted by Cesar Cordeiro), Cayo Arcas (five speakers, in Spanish, hosted by Eduardo Cuevas), and the Mesoamerican Reef (11 speakers, hosted by Healthy Reefs for Healthy People). On average, 127 people watched these live-stream regional sessions and 256 people viewed each recording. Furthermore, Cordeiro and his team hosted a 3-day sub-event in Portuguese, Encontro Recifal Brasileiro, with its own YouTube channel, website, and social media marketing (Box 1).

**Box 1. Cesar Cordeiro (Universidade Federal do Rio de Janeiro) and his team hosted a 3-day sub-event, *Encontro Recifal Brasileiro*, in Portuguese as part of Global Coral Reef Week.**

The Brazilian Reef Meeting, *Encontro Recifal Brasileiro* – EReBra (Portuguese), held as a regionally focused component of GCRW 2020, was the first event dedicated to reef environments in Brazil. The lack of such events in the past has made the local audience seek out international events, involving long distance travel at high economic and environmental costs (Sarabipour et al., 2020 preprint). Traveling is the main cost associated with scientific events for attendees (Rowe, 2019), especially in a large country like Brazil. Being part of GCRW provided the EReBra audience with access to global coral reef research content and a wider network of collaborators. In return, EReBra contributed a live-streamed session focused on technology innovation in reef research to the core GCRW program and access for all GCRW attendees to EReBra's regionally focused recorded talks. EReBra had 1293 registered attendees from all 17 coastal states and five inland states in Brazil. The majority of participants were students (90%), followed by non-academic (4%), academic professionals (3%), and coral reef enthusiasts (3%). While EReBra's regional program was only available in Portuguese, participants were from Portugal, Chile, México, Guatemala, the USA, and Ireland. From a feedback poll, 99% of respondents (*n*=188), rated the event format and content as ‘very good’ or ‘good.’ EReBra was supported by Projeto Costão Rochoso, Projeto Conservação Recifal, Projeto Coral Vivo, & Projeto Budiões.

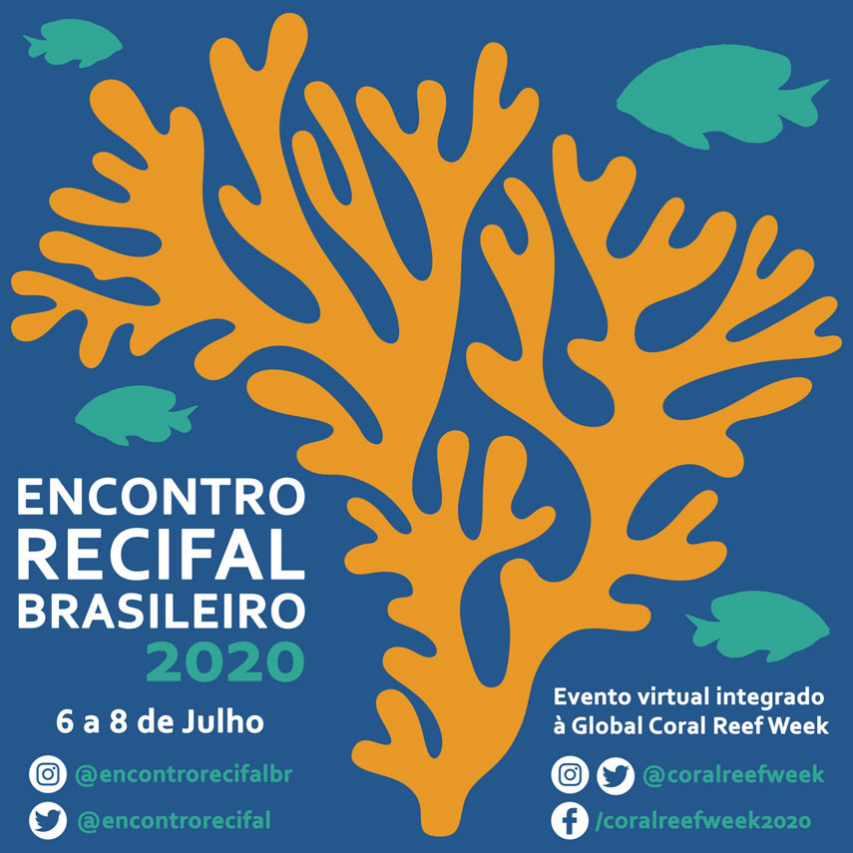

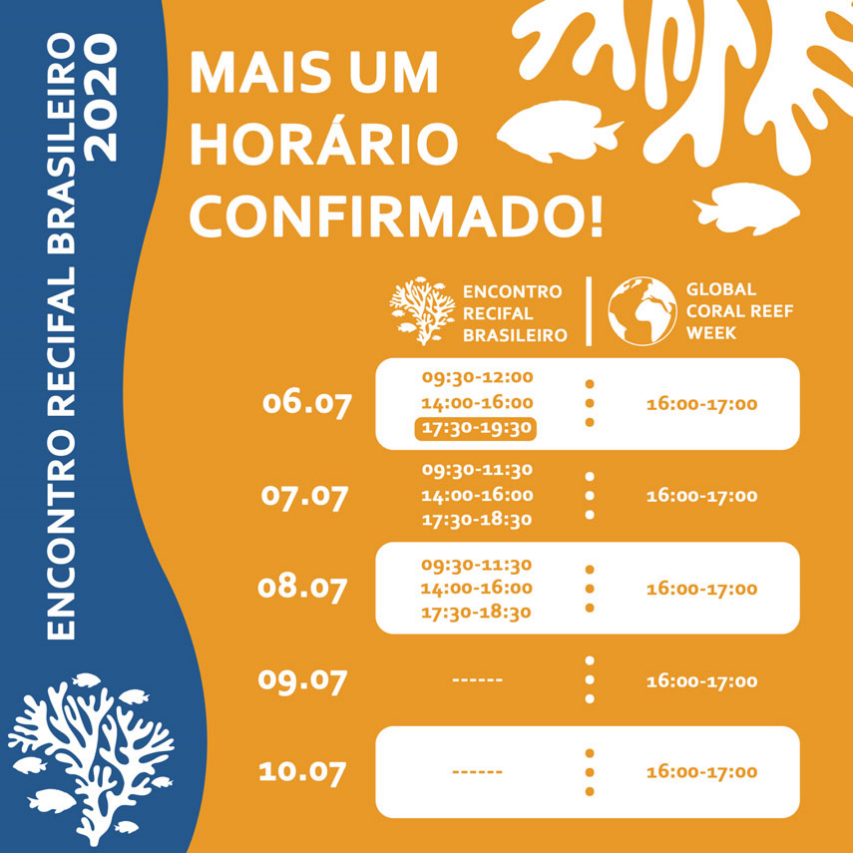

As part of our call for abstracts, we welcomed contributions of virtual workshops. We suggested a recorded format with an introduction, guided activities, and a wrap-up. We received two virtual workshops contributions, one focused on designing and implementing creative ways to combat the climate crisis, from Chelsie Counsell, Hawai‘i Institute of Marine Biology, which received 223 views by the end of GCRW, and another designed in four sequential episodes from Alizee Zimmermann, Turks and Caicos Reef Fund, showing citizen scientists how to identify the coral species susceptible to Stony Coral Tissue Loss Disease, which received an average of 56 views per episode. In response to participant requests shared during GCRW, conference organizer Franziska Elmer partnered with plenary speaker Robby Thigpen (Marine Conservation without Borders) and participant Neus Figueras (University of Barcelona) to lead a live workshop on ways to strengthen outreach and communication. Forty-four conference participants attended this live workshop and the recording had an additional 27 views.

For social activities, GCRW included two networking sessions and a trivia event. The networking sessions included rotation through breakout rooms and icebreaker questions for a total of 49 participants. Our virtual trivia event, led by a team of research interns from the School for Field Studies’ Center for Marine Resource Studies, had 54 registered participants. While casual networking is a challenging element of conferences to recreate virtually, our participants provided positive feedback for these sessions, noting that much of the challenge of inserting oneself into an ongoing in-person conversation was removed. Excitingly, enough science and ideas were shared at GCRW to catalyze a new research collaboration. Building from live-streamed plenaries and conversations in networking sessions, participant Tatiana Becker (Wageningen University) and plenary speaker Phillip Dustan (College of Charleston) are leading a global effort to study the effects of the return of tourism on reefs when COVID-19 travel restrictions are lifted. To date, GCRW has hosted six meetings, providing digital and administrative support for the 30 collaborators participating in this project.

A week before the conference, 167 people had subscribed to GCRW. This number increased to 639 people by the day before GCRW and to 2700 people at its end. The incredible jump in email subscriptions for an inaugural research conference not affiliated with any specific program or society demonstrates the ease with which digital content can be shared across social networks (Box 2).

**Box 2. Madyson Miller (Master's student at University of the Virgin Islands) managed the Instagram, Twitter, and Facebook accounts for Global Coral Reef Week to promote the event.**

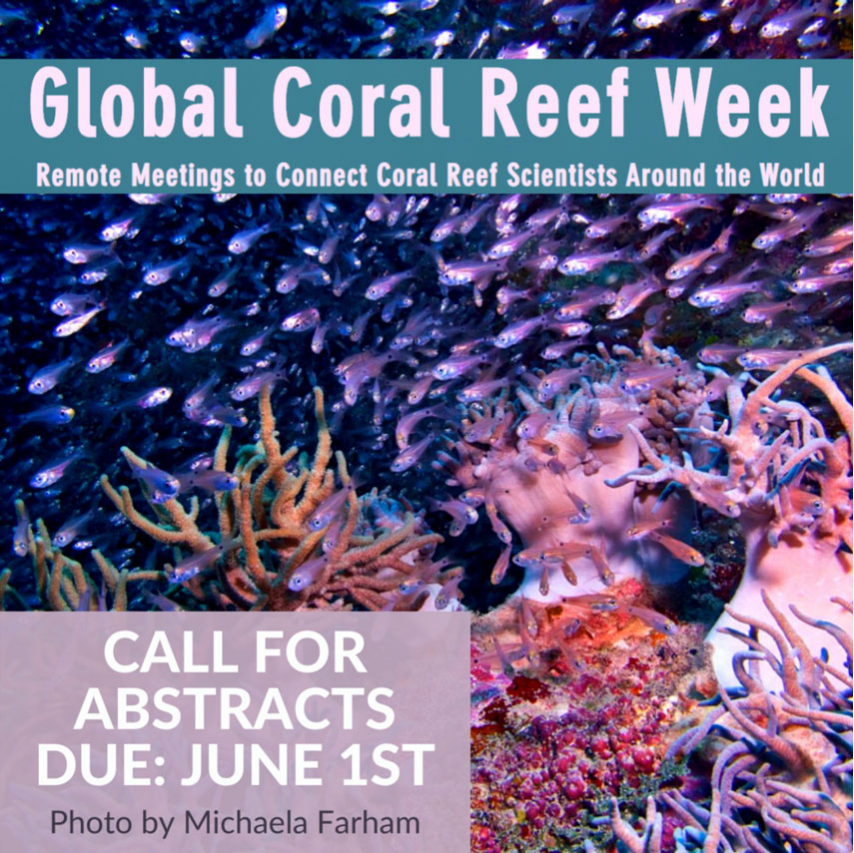

Building a social media presence is crucial for promoting research conferences. For GCRW, we used three different platforms: Instagram (609 followers), Twitter (371 followers), and Facebook (674 followers). To create content, we built a photo bank by reaching out to scientists and photographers around the globe, and then used Canva to design promotional graphics. Posts included conference details (abstract deadlines, subscription information, program scheduling), interesting facts about coral reefs, and details on the research of GCRW participants. To increase interest in our educational content as a mode of informal outreach, we used creative hashtags e.g., #WeirdFactWednesday, #SharkySunday, and #FunFactFriday. Our content also included Instagram ‘takeovers’ where invited scientists shared photos and brief summaries of their research through posts on the GCRW account. On all three social media platforms, people interested in GCRW shared and promoted the conference with their networks by re-posting our content. This chain reaction expansion in reach greatly boosted our audience. On Facebook, >20,000 individuals saw our posts in their feed from 24 June to 19 July 2020.

## Added benefits of the virtual conference format

While GCRW was designed to decrease conference CO_2_ emissions, we quickly recognized a multitude of other benefits of a global virtual research conference. The public, digital archive of content increases access compared to an in-person conference by eliminating scheduling conflicts, enabling rewinding, pausing and re-watching, facilitating content sharing, and providing anyone with internet access an opportunity to learn about current coral reef science. The increased accessibility of digital content is of particular importance for participants who are experiencing personal life events or have certain disabilities that make travel to conferences unfeasible. For example, an attendee of GCRW had just finished cancer treatment, yet was able to join our virtual conference and even to contribute a presentation. Conference content in the form of YouTube recordings provides access to researchers who are deaf or who have limited understanding of the language through auto-translation subtitles. When contributors choose to keep their videos available online, these benefits of increased access to virtual content continue beyond the end of the conference. Broad sharing of science with current or future colleagues, employers, advisors, and students, as well as resource managers, politicians, the media, and community partners is enabled.

Hosting a research conference on virtual platforms affords additional flexibility for programming and content compared to in-person conferences. Without temporal and spatial restrictions on the number of conference rooms, we could accept all topically relevant research contributions. We were able to adjust the thematic sessions, i.e., YouTube playlists, to match the interests of conference participants revealed in their contributed talks. Although we asked for 3–5 or 12–15 min presentations, when presenters took an additional minute or ten, the talks could be included in their entirety without disrupting the conference schedule. Alternatively, when a subset of contributed abstracts did not result in video submissions, these gaps in content were easily removed from our digital program. No attendees arrived expecting a speaker, only to find an empty room. Another powerful aspect of high flexibility in virtual programming is the ability to respond to participant requests in real time. We deliberately did not schedule live events on the last two conference days, which enabled us to organize and host additional programming (i.e., live-streamed workshops and collaborative research-project-development meetings) in response to the expressed interest of our participants.

Virtual conferences incorporate a variety of health benefits for attendees as a result of minimizing long distance air travel. Personal benefits of flying less include reduced exposure to germs and misalignment of the circadian rhythm. Jet lag affects gene expression connected with aging processes and immune system functioning (Cohen and Kantenbacher, 2020). Chronic jet lag can lead to memory impairment and increase the risk of stroke and heart attack (Cohen and Kantenbacher, 2020). In addition, flying exposes travellers to radiation at exposure levels hundreds of times higher than other travel options and chronic exposure to engine noise at levels within passenger cabins is linked to increased risk of heart disease and hearing loss (Cohen and Kantenbacher, 2020). The health impacts of traveling to conferences can also include social strain from leaving families and stress from postponing other work activities (Høyer and Naess, 2001).

The cost benefits of GCRW were enormous. In-person conference organizers with a similar number of speakers to GCRW need to raise around $20,000 to $60,000 for non-labor related costs, whereas our non-labor costs were <$1000 (all estimates in USD). In-person conference attendance can cost participants >$1000; registration for GCRW was free and participation did not require travel or lodging. Researchers from diverse social-economic backgrounds attended GCRW, including those who are unable to afford in-person conferences. GCRW cost <$10 per presenter and <$0.50 per attendee, expenses that were covered through the support of a grant, sponsors, and donations. In terms of the time required of the organizers, this virtual experience was similar to in-person conferences. However, the time commitment will likely be reduced for future GCRW events as it included concept development, building a website, familiarizing ourselves with the capacity of various virtual platforms, and writing guides to help participants navigate the virtual milieu.

## Steps to organize a virtual research conference

To guide others who are interested in organizing research conferences with virtual components, we have documented the key steps required to host a meeting like GCRW along with a timeline that reflects our process. In total, >530 h were required to organize GCRW.

1 Clarify event-specific details (>6 months before conference, 90+ h of organizer time). This includes selecting the event name, creating a logo, defining a target audience, scoping potential research themes, selecting event dates, and building a website (we created a Wix website linked to a Google domain – www.coralreefweek.org). Assessing finances and technological support capacity can help guide platform selection: for live-streaming content we used Zoom, for sharing recorded videos and creating a digital archive we used YouTube, for developing a mailing list to share content links and updates we used Wix built-in tools and Mailchimp, and for promoting the event on social media we used Instagram, Twitter, and Facebook (Box 2).
2. Develop core meeting content (4 months before conference, 40+ h). Invite and confirm participation for plenary speakers. Develop plans for social and networking events, include protocols alerting participants how their contact information may be used (e.g., for email updates) or shared (e.g., with fellow participants). Strategize effective techniques for virtual workshops and begin conversations with potential workshop hosts.
3. Marketing/outreach to solicit for contributed content and conference attendees (start as soon as possible, 40+ h). We created a Google form to collect abstract submissions for contributed talks and workshops. The link to this form and a subscribe option, for people interested in attending the conference without contributing content, were provided on our website. We started marketing the event on email lists for our affiliate research organizations. We contacted individuals acting as local organizers for remote hubs and/or research sessions regarding support needs and programming ideas. We sent newsletter and email templates to local organizers for marketing the event. We used social media accounts to market the conference and abstract submission deadline.
4. Conference administration (1–4 months before conference, 50+ h). Even if content is virtually collected using built-in platform tools, organizers will need to curate the contributions. It is also critical to maintain regular communication and logistical organization with plenary speakers and local partners.
5. Technological support (2–12 weeks before conference, 40+ h). Confirm platforms to be used and obtain rights for their use (we invested in a Zoom Pro account with webinar extension to increase participant access). Solicit volunteers to help moderate content. Train organizers and volunteers on virtual platforms (for us, this included Zoom's back-end tools). Create an abstract book with search capacity so that conference attendees can find content based on key words and author names.
6. Event management (during and after the conference, 190+ h over 14 days). During all live-streamed content, have at least one trained person scheduled to help with IT support. A separate moderator is needed to help enforce time constraints and guide the questions and answers. Continue to curate recorded materials, add recordings of live events, and add links to the website. Use social media platforms to post highlights from the conference and to engage with conference participants. Draft and send emails to conference subscribers with daily updates on available content. Reply to emails from participants. Hold practice sessions with speakers as needed.
7. Funding (start as soon as possible, 30+ h). Evaluate the costs of organizing and supporting your conference. Solicit appropriate funding.

## Future directions

Our vision for a conference of virtually connected, in-person regional hubs would build on the success of the inaugural GRCW. In addition to low CO_2_ emissions, low cost, and high accessibility, remote hubs would provide additional opportunities for face-to-face interactions with local colleagues, educators, students, policy makers, politicians, and the media, greatly strengthening the dissemination of science. Each hub could incorporate a day of environmental or societal service to strengthen engagement with local communities. Virtual connections across the remote hubs would enable participants to network with global community members. We remain energized by local organizers who volunteered to host remote events in 16 international locations and hope someday to realize this vision.

Completely free participation for all attendees of GCRW was possible because of the copious volunteer time contributed (in particular from Elmer and Counsell), a grant from The Company of Biologists, institutional infrastructure support, donations, sponsorships, and merchandise sales. However, there may be good reasons to charge a small conference fee for future events. Attendees and participants may be more actively engaged in paid events to get ‘value’ from their investment. The funds raised could be used to outsource components of the conference organization process and to provide support for virtual platform software and in-person meeting venues.

Without prior experience and without everyone having met face-to-face, we saw a way to provide an option for a collective shift in professional behavior, and we worked hard to make our vision a reality. Along with many others during this difficult pandemic year, we took quick action to create a successful virtual research experience for our colleagues. We have shown that virtual conferences have many benefits and strong potential for growth. To solve the climate crisis, we must embrace creative new approaches for achieving our personal and professional goals with dramatically reduced CO_2_ emissions.
